# Dissecting Heterosis During the Ear Inflorescence Development Stage in Maize *via* a Metabolomics-based Analysis

**DOI:** 10.1038/s41598-018-36446-5

**Published:** 2019-01-18

**Authors:** Xia Shi, Xuehai Zhang, Dakun Shi, Xiangge Zhang, Weihua Li, Jihua Tang

**Affiliations:** 1 National Key Laboratory of Wheat and Maize Crop Science, Henan Agricultural University, Zhengzhou, 450002 China; 2 Hubei Collaborative Innovation Center for Grain Industry, Yangtze University, Jingzhou, 434025 China

## Abstract

Heterosis can increase the yield of many crops and has been extensively applied in agriculture. In maize, female inflorescence architecture directly determines grain yield. Thus, exploring the relationship between early maize ear inflorescence development and heterosis regarding yield-related traits may be helpful for characterizing the molecular mechanisms underlying heterotic performance. In this study, we fine mapped the overdominant heterotic locus (*hlEW2b*), associated with ear width, in an approximately 1.98-Mb region based on analyses of chromosome segment substitution lines and the corresponding testcross population. Maize ear inflorescences at the floral meristem stage were collected from two inbred lines, one chromosome segment substitution line that carried *hlEW2b* (sub-CSSL_16_), the receptor parent lx9801, and the Zheng58 × sub-CSSL_16_ and Zheng58 × lx9801 hybrid lines. A total of 256 metabolites were identified, including 31 and 24 metabolites that were differentially accumulated between the two hybrid lines and between the two inbred lines, respectively. Most of these metabolites are involved in complex regulatory mechanisms important for maize ear development. For example, nucleotides are basic metabolites affecting cell composition and carbohydrate synthesis. Additionally, nicotinate and nicotinamide metabolism is important for photosynthesis, plant stress responses, and cell expansion. Moreover, flavonoid and phenolic metabolites regulate auxin transport and cell apoptosis. Meanwhile, phytohormone biosynthesis and distribution influence the cell cycle and cell proliferation. Our results revealed that changes in metabolite contents may affect the heterotic performance related to ear width and yield in maize hybrid lines. This study provides new clues in heterosis at the metabolomics level and implies that differentially accumulated metabolites made distinct contributions to the heterosis at an early stage of ear inflorescences development.

## Introduction

Heterosis refers to the phenomenon that hybrids perform better than their parents regarding phenotypic traits, including biomass, development rate, fertility, nutrient quality, disease resistance, and crop yield^1,2^. Heterosis can increase grain yield and has been applied to improve agricultural production for a few hundred years. Three classical genetic hypotheses (i.e., dominance hypothesis, overdominance hypothesis, and epistasis hypothesis) were proposed early last century to explain the genetic basis of heterosis. The overdominance hypothesis has been validated using the *SINGLE FLOWER TRUSS* (*SFT*) locus of tomato^3^. However, these hypotheses did not end the century-long debate regarding whether they fully explain the genetic basis of heterosis because they represent largely conceptual models and do not describe the underlying molecular mechanism. Advances in high-throughput technologies have enabled researchers to develop new strategies for dissecting the genetic or molecular mechanism regulating heterosis. However, multiple omics-based researches (i.e., genomics, transcriptomics and epigenetics) in plants for hybrids and their parents, have been conducted to explore the cause of heterosis, the genetic mechanism mediating heterosis remains unclear up to now^4–6^.

Genomics-based studies revealed that the complementation of two parental genomes makes an important contribution to heterosis^7,8^. Moreover, based on transcriptomics and proteomics, additive and non-additive effects have been estimated between parents and hybrids^9,10^. Due to a large amount of redundant information was generated along with the high-throughput data obtained by genomics, transcriptomics and proteomics technologies, making it even more difficult to elucidate the genetic mechanism of heterosis at one or multiple loci. As so far, at the single-locus level, *SFT* is the first identified overdominance gene associated with heterosis, has been cloned in tomato^3^. Although considerable efforts have been made to dissect the genetic basis of heterosis in plants, there is no appropriate method for analyzing heterotic performance because of the complexity of genetic backgrounds and controversies regarding the underlying mechanism.

To eliminate the influence of genetic backgrounds, backcross populations of chromosome segment substitution lines (CSSLs) were developed and used for characterizing the relationship between heterosis and grain yield as well as the associated components in tomato and rice^11,12^. Compared with other populations, such as recombinant inbred lines (RIL) or doubled haploid populations, CSSLs enable the identification of the chromosomal segment responsible for heterotic effects under the same genetic background in multiple environments. Testcross populations have been widely applied to detect quantitative trait loci associated with heterosis^13,14^. For example, Wang *et al*.^12^ used 66 testcross F_1_ lines developed from the corresponding CSSLs to demonstrate that partial dominance and overdominance represent the genetic basis of the heterosis of yield and yield-related traits in rice hybrids. Using a set of 202 CSSLs and the corresponding testcross population, 15 heterotic loci (HLs) for plant height were detected in rice^13^. Integrating the backcross and testcross populations of CSSLs may clarify the genetic and biological basis of heterosis.

Recent metabolomics-based investigations have more thoroughly clarified the metabolic activities of many crops, with potential implications for breeding new high-yielding varieties with improved nutritional composition. Metabolites are useful for efficiently predicting the hybrid performance of several crops, including maize and rice^15,16^. However, relatively few studies comparing the concentrations of secondary metabolites in hybrids and their parents have been conducted in maize, especially involving two inbred lines (one origin inbred line and its progeny with a single chromosome segment from a backcross), with the corresponding hybrids crossed with a common test parent.

Heterosis of ear traits has significant positive correlations with maize yield^17^. Ear traits including ear length, ear width, kernel row number and kernel number per row, are determined in developing immature maize ears^18,19^. During ear inflorescence development, the floret meristem (FM) stage is critical for ear development and heterosis^20^. Consequently, this stage is ideal for studying heterosis. Furthermore, metabolites represent the basic compounds accumulating in plant organs that influence the phenotype. However, there is very little or no available information regarding the relationship between secondary metabolites and heterosis. Thus, analyzing the secondary metabolites in hybrids and their parents may provide new insights and clarify the genetic mechanism underlying heterosis. In this study, we collected immature maize ears during the FM stage from two inbred lines [i.e., sub-CSSL_16_ containing an heterotic locus (HL) associated with ear width and its receptor parent lx9801] and their corresponding hybrids crossed with a common tester, Zheng58. We integrated the results of our targeted metabolomics approach with relevant gene expression data to (1) explore the metabolic processes involved in early maize ear development; (2) identify metabolites associated with heterosis; (3) construct a metabolites interaction network explaining heterotic performance. These results may help to elucidate the mechanism underlying heterotic performance at the metabolomics level.

## Results

### Fine mapping the heterotic locus *hlEW2b*

**Figure 1:**
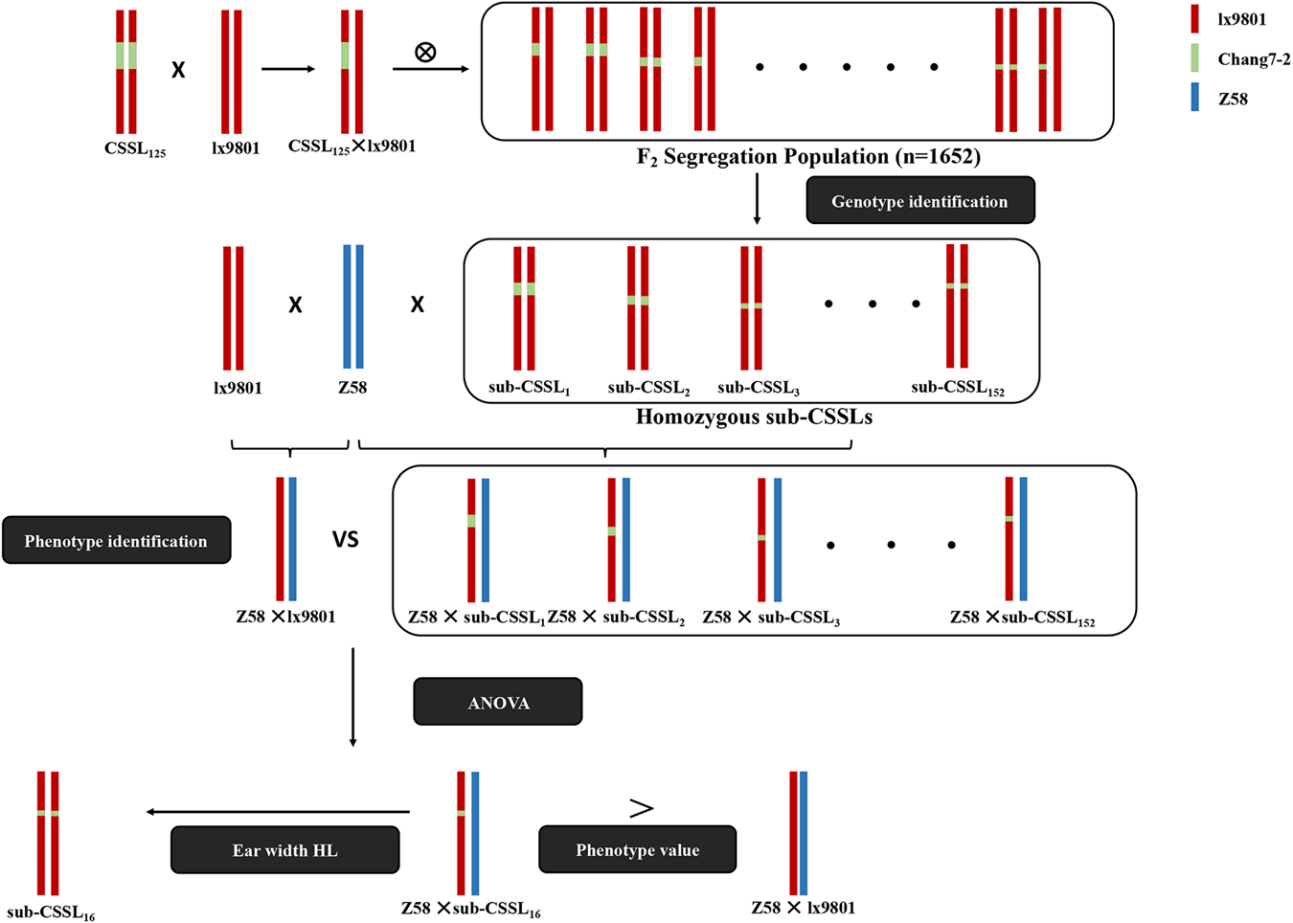
Development of a CSSL testcross population and identification of heterotic loci. The Zheng58 × CSSL_125_ population exhibited significant heterosis for maize ear width. The sub-CSSL population was constructed from 152 homozygous sub-CSSL F_2_ plants, which were derived from a cross between CSSL_125_ and lx9801. The corresponding test population was constructed by crossing the test parent Zheng58 with sub-CSSLs. Red, green, and blue represent the genomes of lx9801 (receptor parent), Chang7-2 (donor parent), and Zheng58 (test parent), respectively.

We previously mapped *hlEW2b* on chromosome 2 between markers umc1555 and umc1465, and determined that it accounts for 8.5% of the ear width heterosis over the standard control (Zheng58 × lx9801)^21^. To more precisely locate *hlEW2b*, 152 homozygous sub-CSSLs were identified among 1,652 F_2_ individuals derived from the CSSL_125_ × lx9801 cross by genotyping with 12 new polymorphism markers within the *hlEW2b* region. Meanwhile, a test population was generated by crossing 152 homozygous sub-CSSLs with the test parent Zheng58 (Fig. 1). We compared the ear width of Zheng58 × lx9801 with that of the test population. A total of 37 homozygous recombinant lines carrying the *hlEW2b*-containing donor genomic segment between SSR markers 7HY-304 and umc1024 had ears that were wider than those of Zheng58 × lx9801 (P < 0.01). Screening with molecular markers resulted in the mapping of *hlEW2b* to a 1.98-Mb region between 7HY-304 and 1HY-100 (see Supplementary Fig. S1).

A total of 42 candidate genes, including 30 functional genes coding proteins, 3 transcription factors and 8 unknown functional genes, were found in this mapping region (Supplementary Table S1). Three genes, Zm00001d002896, Zm00001d002929 and Zm00001d002934 encodes TUB-transcription factor, auxin response factor and NAC domain-transcription factor, respectively. Additionally, Zm00001d002882 encodes SKP1-interacting partner and Zm00001d002933 encodes DDB1-, CUL4-associated factor has the same functions, all of which are component of the E3 ubiquitin-protein ligase complex^22^. One genes Zm00001d002888 [encoding Medicago truncatula NODULIN 21 (MtN21)] was a homolog of Zm00001d002893 [encoding Walls-Are-Thin 1 (WAT1) related protein], which could disrupt auxin transport to regulating cell elongation^23^. Moreover, several candidate genes encode some important enzymes, for example, Zm00001d002880 encodes an isopropylmalate dehydrogenase, Zm00001d002889 encodes a soluble inorganic pyrophosphatase, Zm00001d002922 encode an inositol polyphosphate multi kinase beta, Zm00001d002912, Zm00001d002916 encode the glyoxal oxidase-related protein and five candidate genes (Zm00001d002897, Zm00001d002898, Zm00001d002899, Zm00001d002901 and Zm00001d002902) encode the peroxidase. These candidate genes maybe contribute to heterosis, more work needed to narrow down the mapping interval and confirm the functional gene. Furthermore, according to the heterosis for ear width and the genotype of the homozygous recombinant lines, one sub-CSSL_16_ line with the shortest donor chromosomal segment (1.98 Mb) harboring *hlEW2b* was selected for a metabolomics analysis.

### Phenotype and heterosis effects analysis

Phenotypic analyses in four environments revealed significant differences in ear width (P < 0.01) between the hybrids of Zheng58 × sub-CSSL_16_ and the Zheng58 × lx9801, while there were no significant differences in other ear related traits (including ear length, kernel row number and kernel number per row) (Table 1). Additionally, there were no significant differences in all five ear traits between the inbred lines lx9801 and the sub-CSSL_16_ in two environments (Supplementary Table S2). In order to characterize the genetic effect of HL (*hlEW2b*), we calculated the additive and dominant value of HL (*hlEW2b*) based on the following equations: a = (P_1_–P_2_)/2 and d = F_1_−(P_1_ + P_2_)/2, here, a is the additive effect, d is the dominant effect, P_1_ refers to the trait value of Zheng58, P_2_ stands for the trait value of sub-CSSL_16_, and F_1_ refers to the value of the trait for the hybrid Zheng58 × sub-CSSL_16_). Theoretically, a ratio of estimated dominance to the absolute value of additive effect (d/a) >1, =1, <1 were regarded as overdominance, dominance and partial-dominance, respectively^24^. Our results revealed that the HL (*hlEW2b*) of ear width exhibits high overdominance (d/a»1, d = 4.19, a = 0.07) (Supplementary Table S3). Thus, overdominance is the most contributor to heterosis of ear width.

**Table 1:** Maize ear traits of Zheng58 × lx9801 and Zheng58 × sub-CSSL_16_ in four environments.

Trait	Environments	Hybrids		
		Zheng58 × lx9801	Zheng58 × sub-CSSL_16_	P-value
Ear width(mm)	E1	50.71 ± 1.00	52.70 ± 1.30**	1.17E-03
	E2	46.16 ± 0.96	47.64 ± 1.32*	1.04E-02
	E3	51.24 ± 0.65	53.08 ± 1.11**	2.65E-04
	E4	50.09 ± 1.43	53.12 ± 1.26**	8.62E-05
Ear length (cm)	E1	19.18 ± 0.77	19.25 ± 0.78	8.43E-01
	E2	18.56 ± 1.14	19.10 ± 0.75	2.27E-01
	E3	19.44 ± 0.85	18.86 ± 1.30	9.14E-02
	E4	18.35 ± 1.49	17.58 ± 0.93	1.83E-01
Kernel row number	E1	13.80 ± 1.48	14.40 ± 0.84	2.79E-01
	E2	13.60 ± 1.26	13.40 ± 1.65	7.64E-01
	E3	13.40 ± 1.65	13.20 ± 1.40	7.73E-01
	E4	12.80 ± 1.03	13.20 ± 1.03	3.98E-01
Kernel number per row	E1	36.00 ± 3.02	34.60 ± 1.65	2.14E-01
	E2	38.70 ± 1.16	37.20 ± 2.35	8.68E-02
	E3	38.70 ± 1.89	37.30 ± 2.36	1.60E-01
	E4	37.20 ± 1.99	38.40 ± 2.01	1.96E-01
Ear weight (g)	E1	181.00 ± 22.35	204.00 ± 10.75**	8.87E-03
	E2	118.93 ± 5.79	125.45 ± 6.38*	2.78E-02
	E3	211.00 ± 13.70	225.00 ± 14.34*	3.85E-02
	E4	195.00 ± 13.54	216.00 ± 24.13*	2.74E-02

E1, E2, E3, and E4 represent Xinxiang and Hebi in 2015 and Xinxiang and Hebi in 2016, respectively.

*^,^ ** indicate significant differences at the 0.05 and 0.01 probability levels, respectively.

### Global metabolomics

**Figure 2:**
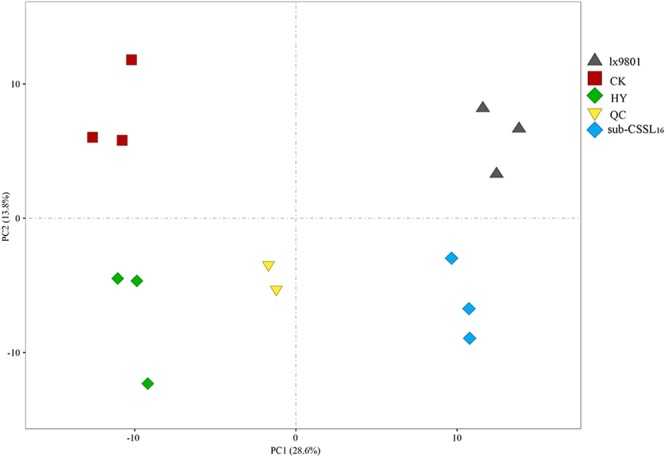
Principal component analysis score plots. The first two principal component (PCs) explained 42.4% of the variation. Additionally, PC1 separated the hybrid lines from the inbred lines, and explained 28.6% of the variation. Meanwhile, PC2 explained 13.8% of the variation, indicating the hybrid lines could be discriminated from the inbred lines. Three biological replicates of each sample were clustered in the same quadrant. HY: Zheng58 × sub-CSSL_16_; CK: Zheng58 × lx9801.

Considerable differences in the secondary metabolite profiles of ears collected at the FM stage were observed (see Supplementary Fig. S2). A total of 256 metabolites were identified in each hybrid and inbred line. A PCA was completed to objectively interpret the metabolomics data for the analyzed hybrids and inbred lines. All samples could be divided into four groups based on the 256 metabolites (Fig. 2). Moreover, 42.4% of the variance in the total data was explained by the first two principal components. An obvious separation between the inbred lines and their corresponding hybrids was detected for PC1, which explained 28.6% of the variance. A clear separation between hybrids was also observed for PC2, which explained 13.8% of the variance. Moreover, two inbred lines, sub-CSSL_16_ and lx9801, were distributed together along the Y-axis. The PCA model also revealed a generally good biological reproducibility within groups because the three biological replicates of each sample exhibited satisfactory clustering. The general trends for Zheng58 × sub-CSSL_16_ and Zheng58 × lx9801 indicated their metabolite profiles were similar. This observation was supported by the fact Zheng58 × sub-CSSL_16_ and Zheng58 × lx9801 were clustered in one subclass (see Supplementary Fig. S2). Moreover, two inbred lines, lx9801 and sub-CSSL_16_, clustered more with each other than with the hybrid lines (see Supplementary Fig. S2). These results implied that the metabolic activities of the hybrids and inbred lines are likely different during the early stage of maize ear development.

### Metabolite-specific accumulation

**Figure 3:**
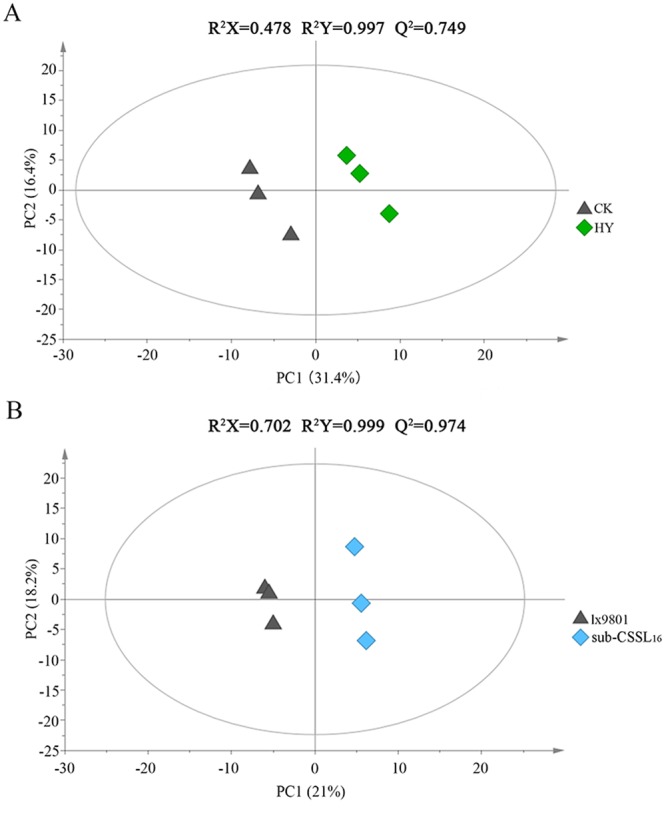
Partial least squares discriminant scores plot. (**A**) Principal component 1 (PC1) and PC2 explained 31.4% and 16.4% of the variation, respectively, indicating Zheng58 × sub-CSSL_16_ (HY) could be discriminated from Zheng58 × lx9801 (CK). (**B**) PC1 and PC2 explained 21% and 18.2% of the variation, illustrating the separation between sub-CSSL_16_ and lx9801. R^2^X, R^2^Y, and Q^2^ refer to the interpretation and predictability values of the partial least squares discriminant analysis models.

Partial least-squares-discriminant analysis (PLS-DA) model is a statistical method for supervised classification that can be exploited to predict the group among new and unlabeled samples^25^. Thus, the PLS-DA supervised multivariate analysis was used to maximize the variance among groups and identify specific metabolites. We applied this model to identify the metabolites that were differentially abundant in the hybrid lines. On the basis of the PLS-DA score plots, we observed an obvious separation between the hybrids regarding the first component (R^2^X = 0.478, R^2^Y = 0.997, Q^2^ = 0.749) (Fig. 3A). This observation indicated the inter-group differences were significant. Additionally, 31 metabolites were differentially abundant between Zheng58 × sub-CSSL_16_ and Zheng58 × lx9801 (Table 2). Moreover, the PLS-DA results for the two inbred lines revealed that sub-CSSL_16_ was markedly different from lx9801 (R^2^X = 0.702, R^2^Y = 0.999, and Q^2^ = 0.974) (Fig. 3B). A total of 24 differentially abundant metabolites were identified based on the VIP and fold-change ratio thresholds (Table 3).

**Table 2:** Identified metabolites significantly differentially accumulated in Zheng58 × lx9801 and Zheng58 × sub-CSSL_16_.

	Compound name	Zheng58 × lx9801	Zheng58 × sub-CSSL_16_	Fold change	VIP^a^	
ALKALOIDS	Papaverine	4.50E + 04	2.88E + 04	0.64	1.38	down
	Trigonelline	3.80E + 05	6.42E + 05	1.69	1.84	up
AMINO ACID DERIVATIVES	Aspartic acid O-rutinoside	7.12E + 05	4.41E + 05	0.62	1.73	down
	Phenylglycine	1.28E + 06	3.21E + 06	2.52	2.17	up
	3-(2-Naphthyl)-D-alanine	3.81E + 04	1.65E + 05	4.33	2.79	up
	Homocystine	2.49E + 06	4.24E + 06	1.70	1.61	up
CARBOHYDRATES	D(-)-Threose	1.07E + 04	1.67E + 04	1.56	1.29	up
	Sucrose	2.31E + 05	4.99E + 05	2.16	2.29	up
CHOLINES	Carbachol	1.41E + 04	2.95E + 04	2.09	2.13	up
COUMARINS AND ITS DERIVATIVES	4-Methylumbelliferone	4.74E + 04	3.00E + 04	0.63	1.39	down
FLAVONOID	Tricetin	1.42E + 05	3.16E + 03	0.02	2.40	down
	C-hexosyl-apigenin C-pentoside	3.76E + 03	2.43E + 03	0.65	1.53	down
	Quercetin O-rutinoside	2.74E + 03	4.24E + 03	1.55	1.52	up
LIPIDS-GLYCEROPHOSPHOLIPID	LysoPC 20:1	4.08E + 05	6.40E + 05	1.57	1.66	up
	LysoPC 20:2	1.40E + 05	2.34E + 05	1.67	1.64	up
NUCLEOTIDE AND ITS DERIVATES	beta-Nicotinamide adenine dinucleotide	3.53E + 05	2.29E + 05	0.65	1.41	down
	Inosine	2.01E + 05	1.33E + 05	0.66	1.48	down
	Guanine	1.09E + 05	1.77E + 05	1.62	1.58	up
	2′-Deoxyadenosine monohydrate	8.56E + 05	1.67E + 06	1.96	1.82	up
	Guanosine 5′-monophosphate	5.23E + 04	1.04E + 05	1.99	1.84	up
	Thymine	1.57E + 05	3.48E + 05	2.22	1.93	up
PHENYLPROPANOIDS	Sinapoyl O-hexoside	8.90E + 04	2.00E + 05	2.25	1.96	up
	Sinapic acid	2.04E + 04	4.81E + 04	2.36	2.25	up
	Geranyl acetate	1.32E + 04	4.74E + 04	3.60	2.95	up
PHYTOHORMONES	Kinetin riboside	4.68E + 04	7.64E + 04	1.63	1.67	up
	trans-Zeatin riboside (tZR)	4.02E + 03	6.98E + 03	1.74	1.82	up
TERPENOIDS	Phytocassane C	9.95E + 03	1.72E + 04	1.73	1.96	up
	Gentiopicrin	9.36E + 03	2.37E + 04	2.53	2.35	up
TRYPTAMINES AND ITS DERIVATIVES	N-acetyltryptamine	1.71E + 03	2.56E + 03	1.50	1.33	up
VITAMINE RELATED	4-Pyridoxic acid O-hexoside	1.16E + 04	1.84E + 04	1.59	1.75	up
	Nicotinamide	3.18E + 03	5.43E + 03	1.71	1.46	up

Variable importance in the projection (VIP) values were obtained from PLS-DA with the threshold of 1.00.

**Table 3:** Identified metabolites significantly differentially accumulated in lx9801 and sub-CSSL_16_.

	Compound name	lx9801	sub-CSSL_16_	Fold change	VIP^a^	
AMINO ACID	N,N-Dimethylglycine	3.78E + 04	2.31E + 04	0.61	1.89	down
	Glu-Glu	3.80E + 04	2.40E + 04	0.63	1.78	down
	Aspartic acid O-rutinoside	5.40E + 05	1.03E + 06	1.91	1.97	up
	L-(−)-Threonine	6.53E + 05	1.00E + 06	1.53	1.51	up
CARBOHYDRATES	D(−)-Threose	8.02E + 03	1.68E + 04	2.09	1.95	up
CHOLINES	sn-Glycero-3-phosphocholine	4.60E + 05	7.07E + 05	1.54	1.02	up
COUMARINS	4-Methylumbelliferone	1.43E + 04	2.38E + 04	1.66	1.79	up
FLAVONOID	Cyanidin 3-O-glucoside	5.78E + 04	7.53E + 03	0.13	3.77	down
	Tangeretin	1.82E + 04	7.40E + 03	0.41	1.81	down
	Tricetin	4.75E + 03	2.63E + 03	0.55	1.03	down
LIPIDS-GLYCEROPHOSPHOLIPID	LysoPC 20:1	4.53E + 05	7.14E + 05	1.58	1.42	up
NUCLEOTIDE	5′-Deoxy-5′-Methylthioadenosine	5.90E + 03	8.86E + 03	1.50	1.53	up
ORGANIC ACID	Ergothioneine	4.72E + 04	2.42E + 04	0.51	2.29	down
PHENOLAMIDES	N-Feruloyl spermidine	5.17E + 03	9.45E + 03	1.83	2.15	up
PHENYLPROPANOIDS	Sinapoyl O-hexoside	1.58E + 05	2.41E + 05	1.52	1.45	up
	Coumarin	3.67E + 04	5.96E + 04	1.63	1.63	up
	Quinic acid	6.39E + 03	1.07E + 04	1.68	1.65	up
PHYTOHORMONES	N-[(−)-Jasmonoyl]-(L)-Isoleucine (JA-Ile)	3.20E + 03	1.04E + 03	0.33	2.52	down
	(+)-Jasmonic acid (JA)	2.14E + 04	1.10E + 04	0.51	2.00	down
	(+)-*cis,trans*-Abscisic acid (ABA)	2.57E + 05	4.29E + 05	1.67	1.87	up
	trans-Zeatin (tZ)	1.88E + 04	3.66E + 04	1.95	2.31	up
TERPENOIDS	Diosgenin	1.75E + 06	2.91E + 06	1.66	2.10	up
VITAMINE	Nicotinate ribonucleoside	1.79E + 05	9.55E + 04	0.53	2.09	down
	(S)-(+)-2-(anilinomethyl)pyrrolidine	1.56E + 04	2.48E + 04	1.59		up

(a) Variable importance in the projection (VIP) values were obtained from PLS-DA with the threshold of 1.00.

**Figure 4:**
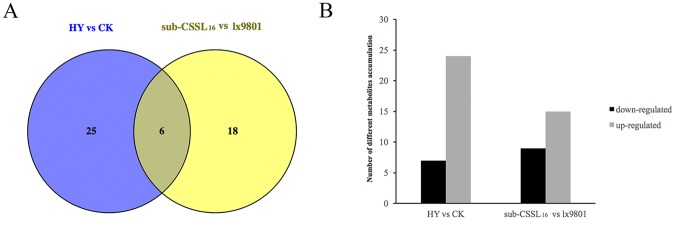
Differentially accumulated metabolites between hybrid and inbred lines. (**A**) Venn diagram of differentially accumulated metabolites in sub-CSSL_16_ and lx9801 as well as in Zheng58 × sub-CSSL_16_ (HY) and Zheng58 × lx9801 (CK). (**B**) Number of differentially accumulated metabolites in hybrids and inbred lines.

A Venn diagram revealed six common metabolites in both comparison groups (i.e., Zheng58 × sub-CSSL_16_ vs. Zheng58 × lx9801 and sub-CSSL_16_ vs. lx9801). Furthermore, 25 metabolites were differentially accumulated between Zheng58 × sub-CSSL_16_ and Zheng58 × lx9801, while 18 metabolites were only differentially accumulated between sub-CSSL_16_ and lx9801 (Fig. 4A). Of all the differentially abundant metabolites, 24 and 15 metabolites were upregulated, while seven and nine metabolites were downregulated in the Zheng58 × sub-CSSL_16_ vs. Zheng58 × lx9801 and sub-CSSL_16_ vs. lx9801 comparisons, respectively (Fig. 4B). Clearly, many metabolites identified in the current study by metabolomics presenting further evidence to understand metabolic processes that might involve in heterosis.

### Secondary metabolite biosynthetic pathway

**Figure 5:**
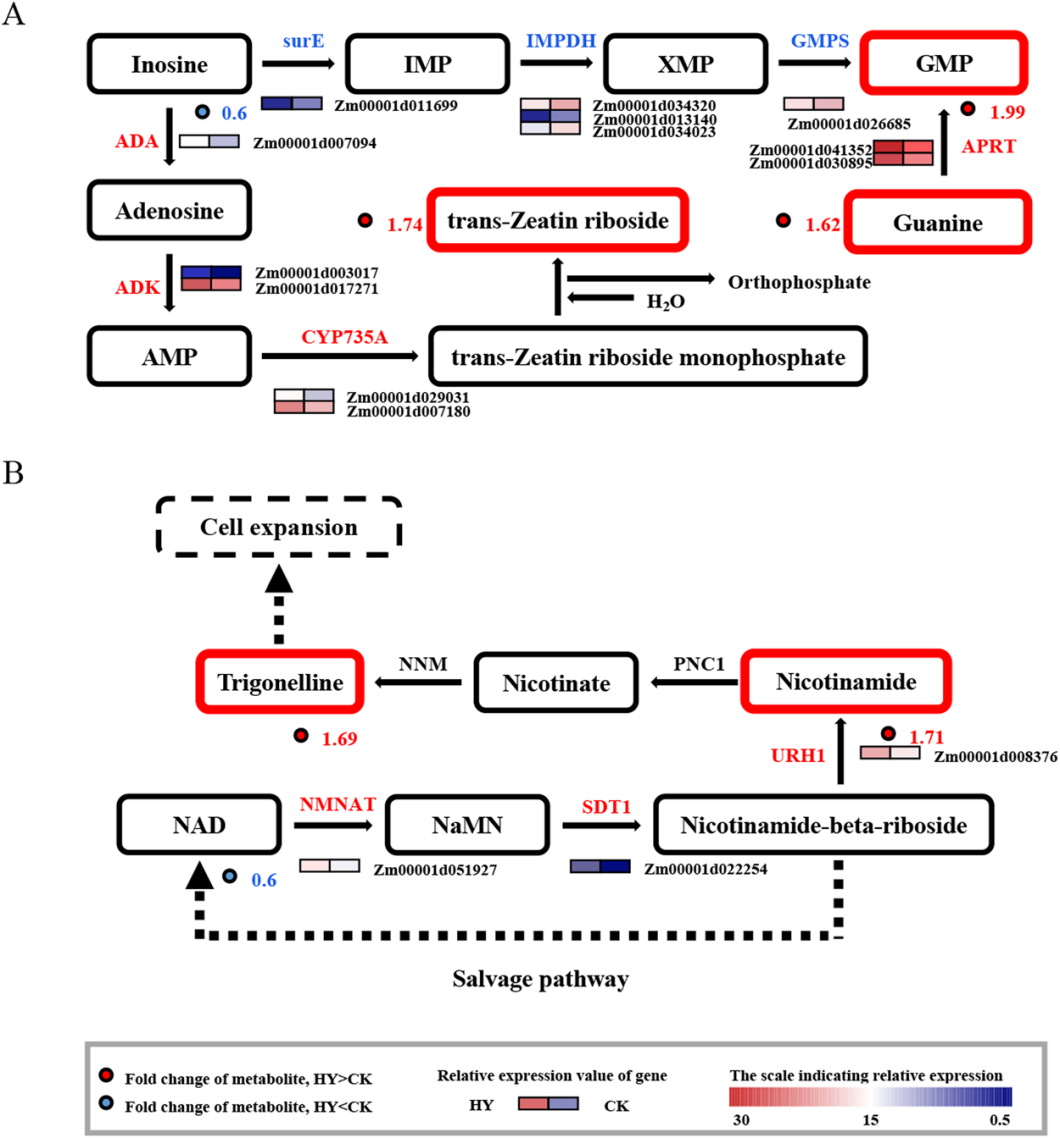
Integrated metabolite changes and differentially expressed genes for analyzing the secondary metabolite biosynthetic pathways. Differentially accumulated metabolites indicated in red and blue were upregulated and downregulated, respectively. The red-to-blue colors in squares indicate high-to-low gene expression levels, respectively. (**A**) surE, 5′-nucleotidase; IMPDH, inosine-5′-phosphate dehydrogenase; GMPS, guanosine 5′-monophosphate synthetase; APRT, adenine phosphoribosyl transferase; ADA, adenosine deaminase; ADK, adenosine kinase; CYP735A, cytokinin *trans*-hydroxylase; (**B**) NMNAT, nicotinamide mononucleotide adenylyltransferase; SDT1, pyrimidine and pyridine-specific 5′-nucleotidase; URH1, uridine nucleosidase; PNC1, nicotinamidase; NNM, nicotinamide *N*-methyltransferase.

To fully characterize the pathways that maybe involve in heterosis during an early stage of ear development, a network was constructed based on differentially abundant metabolites in the two hybrids. In this study, regarding nucleotide metabolism, guanosine 5′-monophosphate (GMP) belongs to purine nucleotide was 1.99-fold higher in Zheng58 × sub-CSSL_16_ than in Zheng58 × lx9801, and guanine as an important compounds of GMP having the same trend was 1.62-fold higher in Zheng58 × sub-CSSL_16_ than in Zheng58 × lx9801 (Fig. 5A). Additionally, under the catalysis of structural genes, the flux of inosine to tZR monophosphate was enhanced in the trans-zeatin riboside (tZR) biosynthetic pathway and tZR monophosphate can be further hydrolyzed to tZR. Consequently, the tZR abundance was 1.74-fold higher in Zheng58 × sub-CSSL_16_ than in Zheng58 × lx9801 (Fig. 5A). In terms of the nicotinate and nicotinamide metabolic pathway, the abundance of nicotinamide showed 1.71-fold increments in Zheng58 × sub-CSSL_16_ than in Zheng58 × lx9801 and nicotinamide as the precursor for synthesizing trigonelline may be also supported the higher accumulation of trigonelline (1.69-fold increments) in Zheng58 × sub-CSSL_16_ than in Zheng58 × lx9801 (Fig. 5B).

### Gene expression pattern analysis

**Figure 6:**
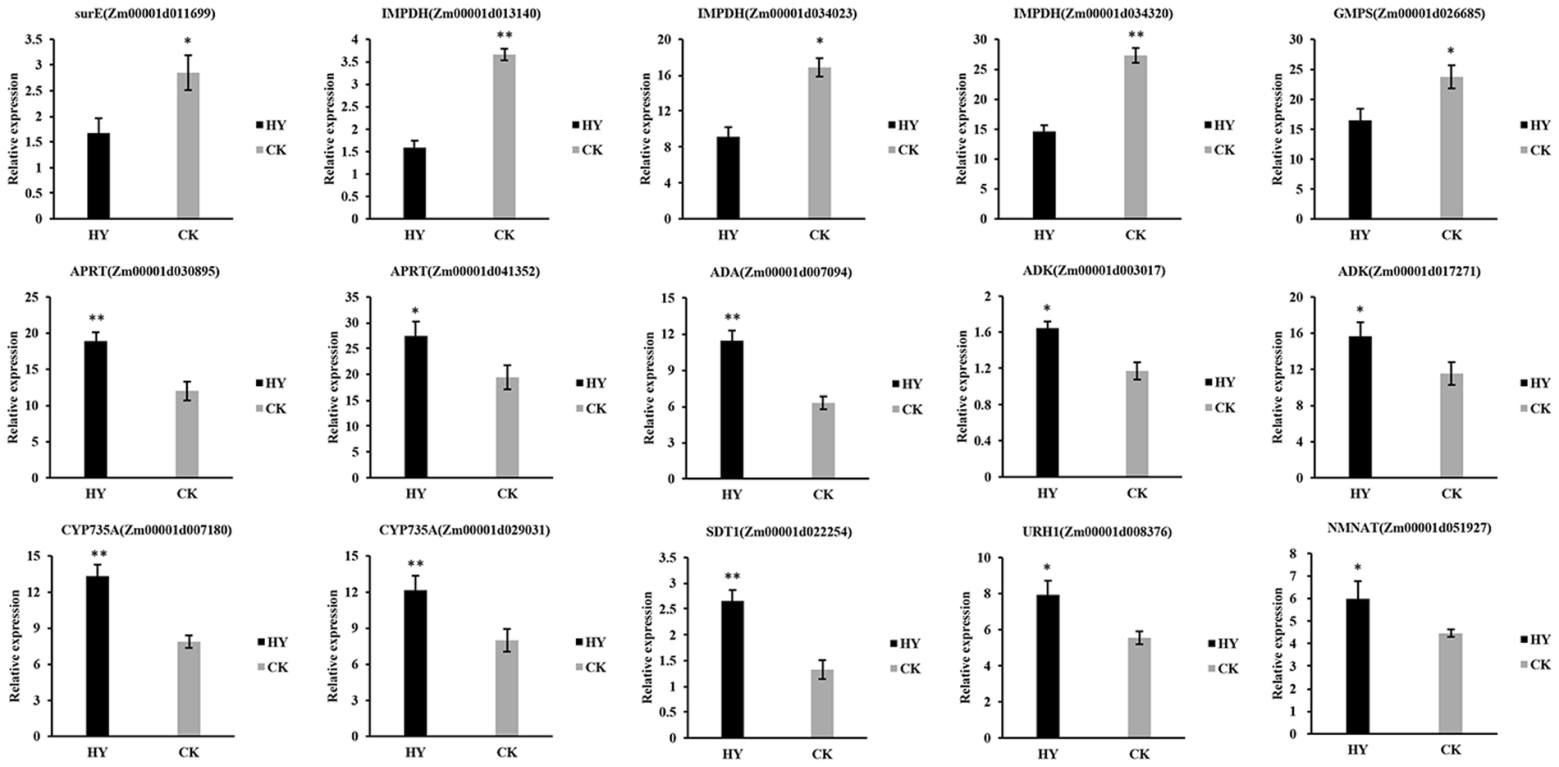
Relative expression levels of genes in secondary metabolite biosynthetic pathways as determined by qRT-PCR. Relative expression levels are presented as the mean of three replicates ± standard error. HY: Zheng58 × sub-CSSL_16_; CK: Zheng58 × lx9801. * and **Indicate significant differences at P < 0.05 and P < 0.01, respectively, as determined by a one-way ANOVA with Student’s *t*-test.

Most secondary metabolic pathways are regulated at the gene expression level. To investigate the expression of key genes, we completed quantitative real-time PCR (qRT-PCR) analyses. A total of 15 candidate genes associated with major secondary metabolic pathways were selected for an analysis of the secondary metabolism related to heterosis during the early stage of maize ear development. During the nucleotide metabolism, the genes of *surE* [5′-nucleotidase; one differentially expressed gene (DEG)], *IMPDH* (inosine-5′-phosphate dehydrogenase; three DEGs) and *GMPS* (guanosine 5′-monophosphate synthetase; one DEG) that involved in the purine metabolic pathway were expressed at lower levels in Zheng58 × sub-CSSL_16_ than in Zheng58 × lx9801 (Fig. 6). These results implied that the conversion of inosine to GMP was less efficiency in Zheng58 × sub-CSSL_16_ than in Zheng58 × lx9801. In contrast, the expression levels of several genes were upregulated in Zheng58 × sub-CSSL_16_, including *APRT* (adenine phosphoribosyl transferase; two DEGs), *ADA* (adenosine deaminase; one DEG), and *ADK* (adenosine kinase; two DEGs) (Fig. 6). Meanwhile, the expression of *CYP735A* (cytokinin trans-hydroxylase; two DEGs), which is involved in zeatin biosynthetic pathways, was significantly upregulated in Zheng58 × sub-CSSL_16_ (Fig. 6). These genes expression pattern were responsible for the increased biosynthesis of tZR in Zheng58 × sub-CSSL_16_.

In the nicotinate and nicotinamide metabolic pathway, the expression of *NMNAT* (nicotinamide mononucleotide adenylyl transferase; one DEG), which is an important structural gene related to nicotinamide mononucleotide (NaMN) synthesis, was significantly upregulated in Zheng58 × sub-CSSL_16_ (Fig. 6). Additionally, *SDT1*(pyrimidine and pyridine-specific 5′-nucleotidase; one DEG) was one of the most significantly upregulated DEGs (P = 5.25E−03) in Zheng58 × sub-CSSL_16_ vs. Zheng58 × lx9801 group. Meanwhile, *URH1*(uridine nucleosidase; one DEG) catalyzing nicotinamide riboside (NR) to nicotinamide was upregulated in Zheng58 × sub-CSSL_16_ (Fig. 6). These results are consistent with our metabolomics analysis that trigonelline contents were higher in Zheng58 × sub-CSSL_16_ than in Zheng58 × lx9801.

## Discussion

### Application of a CSSL test population to investigate the genetic basis of heterosis

Heterosis is considerably influenced by the genetic background and environmental conditions. In previous studies, several primary segregated populations, such as BC_1_, F_2_, and F_2:3_, were used to study the genetic basis of heterosis. However, these populations represent temporary genotypes, making it difficult to obtain accurate phenotypic values over multiple years. To overcome the shortcomings of these populations, several permanently segregated populations, such as introgression lines and immortalized F_2_ (IF_2_) populations, have been developed for identifying heterotic loci^24,26^. Hua *et al*.^27^ identified 44 HLs for grain yield and yield-related components in rice using IF_2_ population that were developed from paired crosses of recombinant inbred lines, while Tang *et al*.^28^ detected 13 HLs for grain yield and its components in maize using the same genetic population. Compared with the primary segregated population and other permanently segregated population, the CSSLs and receptor parent test hybrids used in this study may be more appropriate for dissecting the genetic basis of heterosis. First, the influence of the genetic background can be eliminated during the identification of HLs in comparisons between the test population and the control hybrid Zheng58 × lx9801. Second, accurate phenotypic values may be obtained for the test hybrids under different environmental conditions during the identification of HLs.

Predicting the generation of high-performance hybrids is an integral part of maize breeding. However, only small subsets of potential crosses have been evaluated under field conditions for selecting elite hybrids. Thus, predicting hybrid performance is important for breeding programs. Advanced molecular technologies enabled the acquisition of system-wide genome data for predicting hybrid performance^15^. High-throughput technologies were recently applied to accurately measure the accumulation of metabolites, resulting in large amounts of metabolomics data for analyzing rice hybrid performance^16^. In another study, Lima *et al*.^29^ detected more robust metabolite changes (e.g., organic acids and amino acids) in hybrid lines than in inbred lines. They also observed that a few secondary metabolites could be used as biological markers to predict heterosis. In this study, we conducted a metabolomics analysis with one CSSL and its receptor parent as well as test hybrids to identify nucleotides and their derivatives, alkaloids, flavonoids, phenolics, and phytohormones mediating heterosis for maize ear development. Our data suggested that several important metabolic pathways affect the heterosis for maize ear development.

### Differentially expressed genes associated with nucleotide metabolism and nicotinate and nicotinamide metabolism

Biosynthetic and catabolic enzymes could regulate the pool size of nucleotides and nucleosides^30^. In this study, the enzyme of *surE* participated in the substrate cycles and influence the balance of nucleotides and nucleosides pools at the cellular levels^31^, which have the lower expressed levels in Zheng58 × sub-CSSL_16_ than in Zheng58 × lx9801. Additionally, the enzymes of *IMPDH* and *GMPS* exist in one branch of the guanine nucleotide synthesis and *IMPDH* controlling the gateway to guanine nucleotides for most organism^32^. The conversion of inosine 5′-monophosphate (IMP) to xanthosine 5′-phosphate (XMP) is the first committed and rate-limiting step in guanine nucleotide biosynthesis, which was catalyzed by *IMPDH*. Subsequently, XMP is converted to GMP through the action of *GMPS*^32^. In this study, some genes in the same branch of guanine nucleotide biosynthetic pathway were expressed at lower levels in Zheng58 × sub-CSSL_16_ than in Zheng58 × lx9801, including *surE*, *IMPDH* and *GMPS*. These observations suggested that the conversion of inosine to GMP was less activity in Zheng58 × sub-CSSL_16_ than in Zheng58 × lx9801. Another branch of guanine nucleotide regeneration is the recycling of guanine. The *APRT* belongs to the phosphoribosyl transferases family. Each *APRTs* can reutilize purine bases (such as guanine), being substrates for the synthesis of high-energy nucleotides, and convert them into mononucleotides (such as GMP), instead of nucleotides *de novo* synthesis consuming high-energy^33^. In this branch of guanine nucleotide regeneration, two genes encoding *APRT* were significantly upregulated in Zheng58 × sub-CSSL_16_ comparing with in Zheng58 × lx9801. This could largely explain the high accumulation of GMP in the Zheng58 × sub-CSSL_16_. Moreover, there are two enzymes potentially contribute to the adenosine metabolism in plant, *ADA* and *ADK*. One major route for adenosine salvage may be catalyzed by *ADA* in specific subcellular compartments^34^, and *ADK* as the housekeep enzymes phosphorylate adenosine to adenosine monophosphate (AMP) in *Arabidopsis thaliana*^35^. Moreover, in many organisms, AMP not only as an essential component in purine nucleotide pools, but also as the substrate be catalyzed to produce isopentenyladenine^36,37^. Furthermore, hydroxylation of isopentenyladenine was a key step, which was catalyzed by *CYP735A* in tZR biosynthetic pathway^37^. In this study, some upregulated expression genes involved in the adenosine metabolism including *ADA*, *ADK* and in the trans-zeatin riboside (tZR) biosynthetic pathway including *CYP735A* were recruited in Zheng58 × sub-CSSL_16_, implied that expression pattern of these genes might be responsible for the increased biosynthesis of tZR in Zheng58 × sub-CSSL_16_.

In the nicotinate and nicotinamide metabolic pathway, the expression of *NMNAT*, as the central enzyme to control this conversion of nicotinamide adenine dinucleotide (NAD) to NaMN^38^, was significantly upregulated in Zheng58 × sub-CSSL_16_. In addition, *SDT1* are responsible for production of NR in cells and its activity is correlates with reduced cellular NAD^39^, which was one of the most significantly DEGs between Zheng58 × sub-CSSL_16_ and Zheng58 × lx9801. These two enzymes have significant impact on the cellular NAD homeostasis, which are the regular steps in NAD metabolism^38,39^. Moreover, *URH1*, as an apparent hydrolytic enzyme, makes a greater contribution to the conversion of NR to nicotinamide, and maintains the higher expression levels than other enzymes in this pathway of NR to nicotinamide salvage^40^. The expression of *URH1* was upregulated in Zheng58 × sub-CSSL_16_, which may result in the higher accumulation of nicotinamide in Zheng58 × sub-CSSL_16_ than Zheng58 × lx9801, thus, more trigonelline was synthesized from the nicotinamide.

### Nucleotide and phytohormone metabolism

Purine and pyrimidine nucleotides are precursors for the synthesis of primary and secondary products as well as the building blocks for nucleic acids. In higher plants, GMP can be used as a feedback signal to terminate the pathway responsible for *de novo* guanine nucleotide biosynthesis^35^. Salvage pathways are essential for the biosynthesis of nucleic acids in all organisms. In plants, they also help regenerate nucleotide pools by converting nucleosides to nucleotides, and they contribute to plant cell metabolism and catabolism^41^. To preserve energy, the purine salvage pathway is activated to regenerate guanine from GMP, while consuming only one adenosine triphosphate (ATP)^42^. Guanine nucleotides are important components of nucleotide pools. A small increase in nucleotide pools is accompanied by an increase in carbohydrate content^43^. In the present study, the purine salvage pathway affected the accumulation of GMP and guanine, with higher levels in Zheng58 × sub-CSSL_16_ than in Zheng58 × lx9801. These results may be explained by the fact that carbohydrates, such as sucrose (2.16-fold change) and D-(−)-threose (1.56-fold change), accumulated more in Zheng58 × sub-CSSL_16_ than in Zheng58 × lx9801 (Table 2). Additionally, carbohydrate synthesis might be limited by the nucleotide supply. Moreover, high sugar concentrations may provide sufficient nutrients for vigorous tissue morphogenesis^44^.

The conversion of inosine to AMP occurs by sequential reactions catalyzed by *ADA* and *ADK*^45,46^. A previous study reported that the first step of cytokinin biosynthesis was the isopentenylation of AMP^47^. Thus, AMP is a direct precursor involved in cytokinin synthesis. Cytokinin free bases, such as tZR and isopentenyl adenine, are physiologically active in plant tissues^48^. The disequilibrium distribution of tZR is a key signal for organ-to-organ communication and positively regulates various growth and developmental processes^37^. As the cell cycle progresses, tZR seems to activate cell division during the early part of the S phase^49^. In this study, the tZR differentially accumulated in the two hybrids. Moreover, the upregulated expression of *CYP375A*, which encodes a cytokinin hydroxylase, enhanced tZR synthesis in Zheng58 × sub-CSSL_16_ by releasing active cytokinins^37^, which may be important for cell division and the early stage of maize ear development. These results implied that tZR may contribute to the heterosis of maize ear development.

### Alkaloid metabolism

Nicotinamide is a vitamin B3 precursor that functions as a fundamental mediator of diverse biological processes^50^. In higher plants, including *Arabidopsis thaliana* and *Oryza sativa*, nicotinamide is likely converted to nicotinic acid and salvaged for NAD biosynthesis, which is generally active in vigorously growing cells^51^. Previous studies involving tobacco (*Nicotiana sylvestris*) provided evidence that NAD is an important coenzyme for photosynthesis and plant stress responses^52,53^. Additionally, trigonelline synthesis during seed germination may contribute to the detoxification of surplus nicotinamide^54^. Moreover, trigonelline induces cell expansion, which is an important determinant of grain width^55^. In this study, the abundance of trigonelline (1.69-fold change) and nicotinamide (1.71-fold change) increased in Zheng58 × sub-CSSL_16_ (Table 2). Thus, nicotinamide and trigonelline may be involved in the heterotic performance during the early stage of ear development. These findings implied that nicotinate and nicotinamide metabolism was more active in Zheng58 × sub-CSSL_16_ than in Zheng58 × lx9801.

### Flavonoid and phenolic metabolism

Flavonoids are common and widespread plant secondary metabolites that affect numerous physiological activities, including the negative regulation of auxin transport^56^. Phosphoglycoprotein is an auxin transporter that requires the hydrolysis of ATP^57^. The *PIN* expression pattern and the subcellular localization of the encoded protein are consistent with the fact PIN functions in polar auxin transport^58^. Flavonoids appear to alter PIN cycling, possibly because of induced changes in auxin concentrations via modulated auxin transport directly mediated by phosphoglycoprotein, rather than through an interaction with PIN^59^. Additionally, C-hexosyl-apigenin C-pentoside was detected as a major flavonoid compound, with lower accumulation in Zheng58 × sub-CSSL_16_ than in Zheng58 × lx9801. These results indicated that flavonoids were more abundant in Zheng58 × lx9801 than in Zheng58 × sub-CSSL_16_, thereby inhibiting the transport of indole-3-acetic acid (IAA) and influencing the early stage of maize ear inflorescence development. Moreover, we also detected other flavonoids. For example, tricetin is a flavonoid exhibiting cytostatic properties by inducing the production of endogenous reactive oxygen species involved in apoptosis^60^. Tricetin concentrations were higher in Zheng58 × lx9801 than in Zheng58 × sub-CSSL_16_ (Table 2), but were similar between inbred line lx9801 and sub-CSSL_16_ (Table 3). These results revealed that the elevated levels of intracellular flavonoids adversely affected cell growth and development by regulating apoptosis, with potential implications for heterosis in maize.

Several known phenolic compounds, such as phenylpropanoids and coumarins, have been classified as natural regulators of plant growth, with phenylpropanoids important for responses to abscisic acid (ABA)^61^. Abscisic acid is a major regulator of plant development, including seed dormancy and germination^62^. Sinapic acid is a small natural molecule from the phenylpropanoid family that might be imbibed into seeds, where it is converted into sinapic acid esters that regulate ABA homeostasis and may promote early seed germination in *Arabidopsis thaliana*^63^. In this study, sinapic acid concentrations were more than 2-fold higher in Zheng58 × sub-CSSL_16_ than in Zheng58 × lx9801 (Table 2). Thus, sinapic acid might interfere with ABA homeostasis to induce early maize ear inflorescence growth in Zheng58 × sub-CSSL_16_.

Auxin biosynthesis and transport promote heterosis in *Arabidopsis thaliana*^64^. Regarding auxin distribution, PIN is the crucial factor facilitating polar auxin transport, while SLR/IAA14 is a key mediator of auxin redistribution that is regulated by 4-methylumbelliferone^65,66^. In this study, 4-methylumbelliferone was detected during the early development of maize ear inflorescences. Additionally, 4-methylumbelliferone accumulated more in Zheng58 × lx9801 than in Zheng58 × sub-CSSL_16_ (Table 2), implying that it might restrict the IAA redistribution in Zheng58 × lx9801 and interfere with maize ear growth and development in the FM stage. These results provide new evidence that changes to phenolic compounds influence heterosis in maize.

In this study, variations in gene expression patterns and metabolite contents were analyzed in maize ear inflorescences in CSSL, lx9801, and their corresponding hybrid lines. Additionally, a large-scale targeted metabolomics analysis revealed 24 metabolites that were differentially accumulated between inbred lines, while the contents of 31 metabolites were significantly different in the hybrid lines. The variability in the abundance of metabolites, including nucleotides, alkaloids, flavonoids, and phenolics, as well as in gene expression levels were associated with many biological processes, such as carbohydrate synthesis, cell division and apoptosis, and phytohormone transport and distribution. This study offers novel insights into the influence on secondary metabolism related to heterosis and provides additional information on the mechanism of secondary metabolic pathway involving in heterosis at the metabolomics level.

## Materials and Methods

### Plant materials and the field experiment

Previously, a maize CSSL panel of 184 lines was developed used two maize inbred lines (Chang7-2 as donor parent and lx9801 as the receptor parent), and a corresponding testcross population was constructed by crossing the CSSL panel with a test parent Zheng58. Of the 169 heterotic loci (HL) associated with grain yield were detected in the testcross population of Zheng58 × CSSL after comparing with the control hybrid Ludan9002 (Zheng58 × lx9801)^21^. A maize CSSL_125_ line carrying the HL *hlEW2b* was obtained from one of the CSSL test hybrids (Zheng58 × CSSL_125_) with an ear width significantly different from that of Ludan9002^21^. In this study, various mapping populations, including backcross and inbred populations, were developed in the winter of 2013 (for backcrossing) in Sanya, Hainan province, China (E18°15′, N109°30′) and in the summer of 2014 (for selfing) in Xinxiang, Henan province, China (E113°31′, N35°11′). The sub-CSSL test population was constructed by crossing sub-CSSLs with Zheng58 in the winter of 2014 in Sanya. Additionally, the phenotypes of the sub-CSSL test population and the control hybrid, Ludan9002, were evaluated at two locations, Xinxiang and Hebi (E114°17′, N35°90′), in 2014 and 2015. Plants were grown with two replicates and a randomized complete block design. The control hybrid was also planted after every five testcross plants. Plots consisted of two rows (4 m long and 0.6 m wide) that were separated by 0.22 m. The final plant density was 67,500 plants per hectare. The plants were grown using standard agricultural practices.

### Phenotypic data collection and fine mapping

A maize inbred line, sub-CSSL_16_, which contained the shortest donor chromosomal segment harboring *hlEW2b*, was compared with other homozygous lines in the sub-CSSL population. Two inbred lines, sub-CSSL_16_ and lx9801, along with their corresponding testcrosses, Zheng58 × sub-CSSL_16_ and Zheng58 × lx9801, were planted in two-row plots in four environments (Xinxiang and Hebi in 2015 and 2016). In each plot, 10 ears were harvested at physiological maturity for phenotypic measurements. Ear length (cm), ear width (mm), ear weight (g), kernel row number, and kernel number per row were measured using standard procedures to confirm the presence of *hlEW2b*. Raw data underwent a one-way analysis of variance (ANOVA) using the SPSS 17.0 program (SPSS, Inc., Chicago, IL, USA). The HL associated with ear width were considered to exist in sub-CSSL_16_ when the ear width in Zheng58 × sub-CSSL_16_ differed significantly from that of Zheng58 × lx9801 (P < 0.05).

In this study, we developed molecular markers for fine mapping. The simple sequence repeat (SSR) markers in the region flanked by umc1555 and umc1465 on chromosome 2 were obtained from the IBM 2008 Neighbors maize linkage map (http://www.maizegdb.org/data_center/map). Additionally, SSR sites were identified with the SSR Hunter program^67^. Primers were designed using the Primer 5.0 program (Premier Biosoft International, Palo Alto, CA, USA). Details regarding the primers used for mapping are listed in Supplementary Table S4. Furthermore, possible candidate genes were identified for *hlEW2b* within 1.98-Mb region based on the filtered working gene list of the maize genome downloaded from MaizeGDB (Zea mays. AGPv4.37, http://www.maizegdb.org). Candidate genes were annotated according to InterProScan (http://www.ebi.ac.uk/interpro/scan.html).

### Sample preparation

Two hybrids, Zheng58 × sub-CSSL_16_ and Zheng58 × lx9801, as well as their corresponding inbred lines, sub-CSSL_16_ and lx9801, were planted at the experimental station of Henan Agricultural University (Zhengzhou, China; E113°65′, N34°76′) on June 20, 2016. The ear development stage was confirmed based on the leaf age index and microscopic observation. Immature ears at the FM development stage were collected. Three biological replicates were collected for each sample, which comprised five randomly collected fresh immature ears. All samples were immediately frozen in liquid nitrogen and stored at −80 °C prior to metabolite analyses.

### Metabolite identification and quantification

Samples were freeze-dried in a vacuum and then homogenized using an Retsch MM400 Mixer Mill (RETSCH GmbH, Haan, Germany) with zirconia beads (90 s at 30 Hz). Metabolites were extracted from 100 mg powder with 70% aqueous methanol containing 0.1 mg/L lidocaine. Samples were incubated overnight at 4 °C and then centrifuged at 10,000 × g for 10 min. A 400-µL aliquot of the extract was filtered through an SCAA-104 membrane (0.22 µm pore size; Millipore), and the filtrate was stored in sample bottles for a subsequent analysis by liquid chromatography (LC) and tandem mass spectrometry (MS/MS). The LC-MS/MS analysis was completed with a Shim-pack UFLC Shimadzu CBM30A HPLC system and a 4500 QTRAP ESI–triple quadrupole–linear ion trap mass spectrometer (Applied Biosystems) equipped with an ESI Turbo Ion-Spray interface, which was operated in a positive ion mode and controlled with the Analyst 1.6 program (AB/SCIEX, Framingham, MA, USA).

Metabolites were identified and quantified according to a published protocol^68^. Briefly, the multiple reaction monitoring model was applied to identify the precursor ion of the target material. Many ion fragments were formed after a collision-induced dissociation, and characteristic ions were filtered from the fragment ions. The retention time and mass-to-charge ratio were measured for all detectable ions. Metabolites were quantified according to integral correction and normalization methods for analyzing mass spectra for metabolites in different samples (i.e., peak area evaluation). Metabolites were analyzed qualitatively and quantitatively.

### Metabolite data analysis

Metabolite data were log_2_-transformed before undergoing a multivariate statistical analysis with the SIMCA-P 13.0 program (Umetrics, Umeå, Sweden). The data then underwent an unsupervised principal component analysis (PCA), which is a widely used unsupervised dimensionality reduction method, to visualize general clustering and trends among the samples. To identify differentially accumulated metabolites for each group, the partial least squares discriminant analysis (PLS-DA) was applied to remove irrelevant variables and maximize the variance among samples^25^. The efficiency and reliability of the PLS-DA model was assessed by a cross-validation with R^2^. The predictive performance was described by the Q^2^ model. The variable importance in the projection (VIP) reflected the contribution of each variable to the model. Variables with a VIP value > 1.0 were considered to influence the separation of samples in the PLS-DA score plots^69^. In addition to the multivariate approaches, fold-change ratios were used to assess the significance of each metabolite. A VIP value > 1.0 and a fold-change ratio ≥1.5 or ≤0.67 were set as the thresholds for screening potential metabolites with significant differences in abundance between samples^70^.

### RNA extraction and quantitative real-time PCR (qRT-PCR) analysis

Total RNA was extracted from each sample using the Trizol^®^ Reagent (Invitrogen, Carlsbad, CA, USA) and then treated with DNase I. The quality of the purified RNA (i.e., degradation and contamination) was characterized by 1% agarose gel electrophoresis and with the Nano LabChip kit (Agilent Technologies, Santa Clara, CA, USA). Purified RNA was quantified at OD260nm by using a NanoDrop™ One (Thermo Fisher Scientific, Wilmington, DE, USA). The RNA was then used as the template for a reverse transcription with the PrimeScript™ RT reagent kit with gDNA Eraser (Takara, Japan). A quantitative real-time polymerase chain reaction (qRT-PCR) assay was conducted with the SYBR Premix Ex Taq Kit (Takara) and the BioRad iQ Cycler (Bio-Rad, Hercules, CA, USA) according to the manufacturer′s instructions. The Primer 5.0 program was used to design the qRT-PCR primers, which are listed in Supplementary Table S5. Each sample was analyzed using three biological replicates and three technical replicates. The 2^−ΔΔCt^ method was applied to estimate relative gene expression levels. Additionally, gene expression data underwent a one-way ANOVA with the SPSS 17.0 program. The significance threshold was set as P < 0.05.

## Electronic supplementary material

Supplementary information
