# Supplementary material for: Dissecting Heterosis During the Ear Inflorescence Development Stage in Maize via a Metabolomics-based Analysis
Source: Sci Rep. 2019 Jan 18;9:212. doi: 10.1038/s41598-018-36446-5 (PMC6338801; doi:10.1038/s41598-018-36446-5)
Supplement: Supplementary file 1 — Supplementary information [file 41598_2018_36446_MOESM1_ESM.pdf]

Dissecting Heterosis During the Ear Inflorescence Development Stage in Maize *via* a Metabolomics-based Analysis

Xia Shi[1*], Xuehai Zhang[1*], Dakun Shi[1], Xiangge Zhang[1], Weihua Li[1 +], Jihua Tang[1,2 +]

[1]National Key Laboratory of Wheat and Maize Crop Science, Henan Agricultural University, Zhengzhou 450002, China
[2]Hubei Collaborative Innovation Center for Grain Industry, Yangtze University, Jingzhou 434025, China

[+] Corresponding authors:
Jihua Tang
Tel.: +86-371-56990188
E-mail: tangjihua1@163.com
Weihua Li
Tel.: +86-371-56990188
E-mail: liwh416@163.com

[*] Xia Shi and Xuehai Zhang contributed equally to this work.

SUPPLEMENTAL INFORMATION

This file includes Supplemental Figure S1-2 and Table S1-5.

Supplementary Figure S1. Sequential fine mapping of *hlEW2b*.

Supplementary Figure S2. Hierarchical clustering analysis of metabolomics data.

Supplementary Table S1. Functional annotation of candidate genes in the mapping region of *hlEW2b*.

Supplementary Table S2. Maize ear traits of lx9801 and sub-$CSSL_{16}$ in two environments.

Supplementary Table S3. The heterotic effects analysis for *hlEW2b* on ear width trait in the testcross of Zheng58 × sub-$CSSL_{16}$.

Supplementary Table S4. Molecular markers developed for fine mapping of *hlEW2b* on chromosome 2.

Supplementary Table S5. Primers used for detecting relative gene expression level.

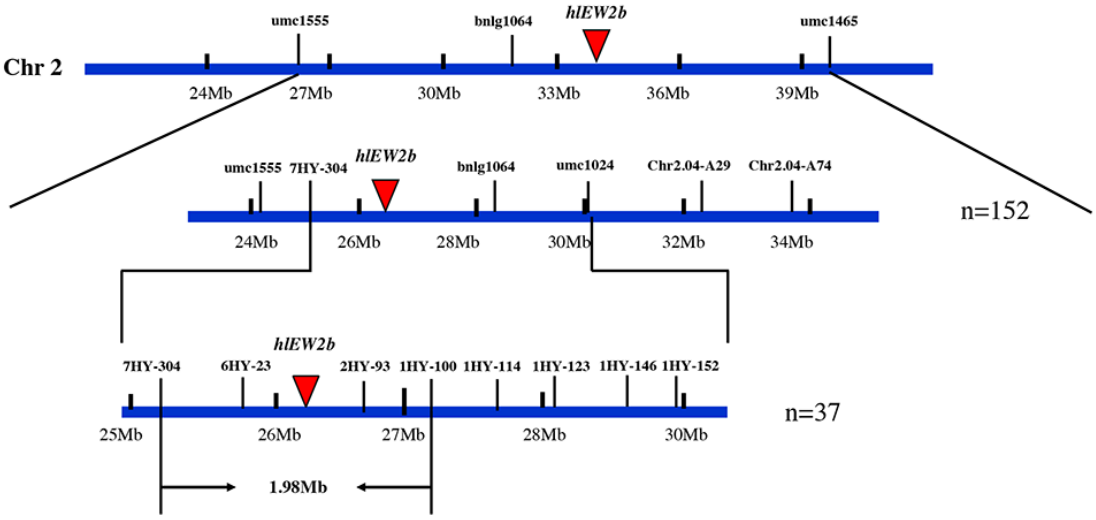

Supplementary Figure S1. Sequential fine mapping of *hlEW2b*. Locations of the fine-mapped regions on chromosome 2 are presented. The *hlEW2b* locus was roughly mapped between the simple sequence repeat markers 7HY-304 and umc1024 on chromosome 2, and subsequently fine-mapped between markers 7HY-304 and 1HY-100, with a physical distance of 1.98 Mb.

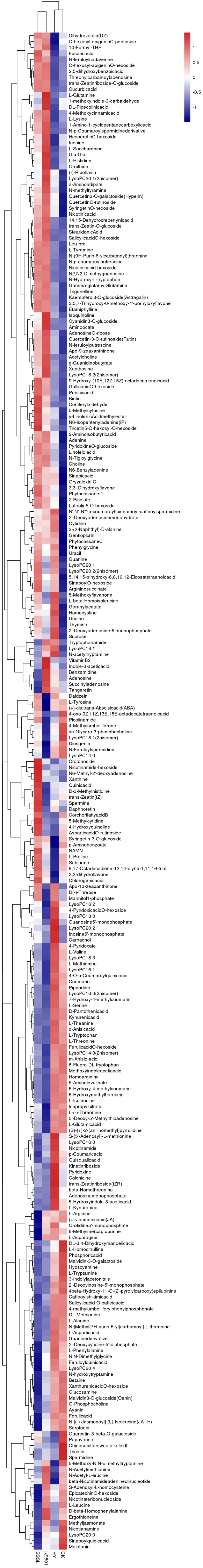
1
0.5
0
-0.5
-1
Dihydrozeatin(DZ)
C-hexosyl-apigeninC-pentoside
10-Formyl-THF
Fusaricacid
N-feruloylcadaverine
C-hexosyl-apigeninO-hexoside
2,5-dihydroxybenzoicacid
Threonylcarbamoyladenosine
trans-Zeatinriboside-O-glucoside
Cucurbicacid
L-Glutamine
1-methoxyindole-3-carbaldehyde
DL-Pipecolinicacid
4-Methoxycinnamicacid
L-Lysine
1-Amino-1-cyclopentanecarboxylicacid
N-p-Coumaroylspermidinederivative
HesperetinC-hexoside
Inosine
L-Saccharopine
Glu-Glu
L-Histidine
Ornithine
(-)-Riboflavin
LysoPC20:1(2nisomer)
a-Aminoadipate
N-methyltyramine
Quercetin3-O-galactoside(Hyperin)
QuercetinO-rutinoside
SyringetinO-hexoside
Nicotinicacid
14,15-Dehydrocrepenynicacid
trans-Zeatin-O-glucoside
StearidonicAcid
SalicylicacidO-hexoside
Leu-pro
L-Tyramine
N-(9H-Purin-6-ylcarbamoyl)threonine
N-p-coumaroylputrescine
Nicotinicacid-hexoside
N2,N2-Dimethyguanosine
N-Hydroxy-L-tryptophan
Gamma-glutamylGlutamine
Trigonelline
Kaempferol3-O-glucoside(Astragalin)
3,5,7-Trihydroxy-6-methoxy-4'-prenyloxyflavone
Etamiphylline
Isoquinoline
Cyanidin3-O-glucoside
Amindocate
AdenosineO-ribose
Quercetin-3-O-rutinoside(Rutin)
N-feruloylputrescine
Apo-9-zeaxanthinone
Acetylcholine
g-Guanidinobutyrate
Xanthosine
LysoPC18:2(2nisomer)
9-Hydroxy-(10E,12Z,15Z)-octadecatrienoicacid
GallicacidO-hexoside
Punicicacid
Biotin
Coniferylaldehyde
5-Methylcytosine
γ-LinolenicAcidmethylester
N6-Isopentenyladenine(iP)
Tricetin5-O-hexosyl-O-hexoside
2-Aminoisobutyricacid
Adenine
PyridoxineO-glucoside
Linoleic acid
N-Tigloylglycine
Choline
N6-Benzyladenine
Sinapicacid
Oryzalexin C
3,3'-Dihydroxyflavone
PhytocassaneD
2-Picolate
Luteolin5-O-hexoside
N',N'',N'''-p-coumaroyl-cinnamoyl-caffeoylspermidine
2'-Deoxyadenosinemonohydrate
Cytidine
3-(2-Naphthyl)-D-alanine
Gentiopicrin
PhytocassaneC
Phenylglycine
Uracil
Guanine
LysoPC20:1
LysoPC20:2(2nisomer)
5,14,15-trihydroxy-6,8,10,12-Eicosatetraenoicacid
SinapoylO-hexoside
Argininosuccinate
5-Methoxyflavanone
L-beta-Homoisoleucine
Geranylacetate
Homocystine
Uridine
Thymine
2'-Deoxyadenosine-5'-monophosphate
Sucrose
Tryptophanamide
LysoPC18:1
N-acetyltryptamine
VitaminB2
Indole-3-aceticacid
Benzamidine
Adenosine
Succinyladenosine
Tangeretin
Daidzein
L-Tyrosine
(+)-cis,trans-Abscisicacid(ABA)
4-oxo-9Z,11Z,13E,15E-octadecatetraenoicacid
Picolinamide
4-Methylumbelliferone
sn-Glycero-3-phosphocholine
LysoPC18:1(2nisomer)
Diosgenin
N-Feruloylspermidine
LysoPC14:0
Crotonoside
Nicotinamide-hexoside
N6-Methyl-2'-deoxyadenosine
Xanthine
Quinicacid
D-3-Methylhistidine
trans-Zeatin(tZ)
Spermine
Daphnoretin
CorchorifattyacidB
5-Methylcytidine
4-Hydroxyquinoline
AsparticacidO-rutinoside
Syringetin-3-O-glucoside
p-Aminobenzoate
NAMN
L-Proline
Sabinene
9,17-Octadecadiene-12,14-diyne-1,11,16-triol
2,3-dihydroflavone
Chlorogenicacid
Apo-13-zeaxanthinone
D(-)-Threose
Mannitol1-phosphate
LysoPC16:2
4-PyridoxicacidO-hexoside
LysoPC18:0
Guanosine5'-monophosphate
LysoPC20:2
Inosine5'-monophosphate
Carbachol
4-Pyridoxate
L-Valine
LysoPC18:3
L-Methionine
LysoPC16:1
4-O-p-Coumaroylquinicacid
Coumarin
Piperidine
LysoPC16:0(2nisomer)
7-Hydroxy-4-methylcoumarin
L-Serine
D-Pantothenicacid
Kynurenicacid
L-Theanine
o-Anisicacid
L-Tryptophan
L-Threonine
FerulicacidO-hexoside
LysoPC14:0(2nisomer)
m-Anisic-acid
6-Fluoro-DL-tryptophan
Methoxyindoleaceticacid
Homoarginine
5-Aminolevulinate
6-Hydroxy-4-methylcoumarin
6-Hydroxymethylherniarin
L-Isoleucine
Isopropylcitrate
L-(-)-Threonine
5'-Deoxy-5'-Methylthioadenosine
L-Glutamicacid
(S)-(+)-2-(anilinomethyl)pyrrolidine
S-(5'-Adenosyl)-L-methionine
LysoPC16:0
Nicotinamide
p-Coumaricacid
Quisqualicacid
Kinetinriboside
Pyridoxine
Colchicine
trans-Zeatinriboside(tZR)
beta-Homothreonine
Adenosinemonophosphate
5-Hydroxyindole-3-aceticacid
L-Kynurenine
L-Arginine
(+)-Jasmonicacid(JA)
Orotidine5'-monophosphate
6-Methylmercaptopurine
L-Asparagine
DL-3,4-Dihydroxymandelicacid
L-Homocitrulline
Phosphoricacid
Malvidin-3-O-galactoside
Hyoscyamine
L-Tryptamine
3-Indolylacetonitrile
2'-Deoxyinosine-5'-monophosphate
4beta-Hydroxy-11-O-(2'-pyrolylcarboxy)epilupinine
Caffeoylshikimicacid
Salicylicacid-O-caffeicacid
4-methylumbelliferylphenylphosphonate
DL-Methionine
L-Alanine
N-[Methyl(7H-purin-6-yl)carbamoyl]-L-threonine
L-Asparticacid
Guaninederivative
2'-Deoxycytidine-5'-diphosphate
L-Phenylalanine
N,N-Dimethylglycine
Feruloylquinicacid
LysoPC20:4
N-hydroxytryptamine
Betaine
XanthurenicacidO-hexoside
Glucosamine
Malvidin3-O-glucoside(Oenin)
O-Phosphocholine
Ayanin
Ferulicacid
N-[(-)-Jasmonoyl]-(L)-Isoleucine(JA-Ile)
Serotonin
Quercetin-3-beta-O-galactoside
Papaverine
ChinesebittersweetalkaloidII
Tricetin
Spermidine
5-Methoxy-N,N-dimethyltryptamine
N-Acetylmethionine
N-Acetyl-L-leucine
beta-Nicotinamideadeninedinucleotide
S-Adenosyl-L-homocysteine
EpicatechinO-hexoside
Nicotinateribonucleoside
L-Leucine
D-beta-Homophenylalanine
Ergothioneine
Methyljasmonate
Nicotianamine
LysoPC20:0
Sinapoylquinicacid
Melatonin
SSSL
lx9801
HY
CK

Supplementary Figure S2. Hierarchical clustering analysis of metabolomics data. The heat map was plotted using the log_2-transformed mean values of metabolomics data.

Supplementary Table S1. Functional annotation of candidate genes in the mapping region of *hlEW2b*.

Chr.	Candidate genes	Annotation [a]
2	Zm00001d002872	Unknown
2	Zm00001d002873	UPF0426 protein chloroplastic
2	Zm00001d002874	OSJNBa0038O10.24 protein
2	Zm00001d002875	Encodes alpha-helical IF (intermediate filament)-like protein.
2	Zm00001d002876	Regulator of chromosome condensation (RCC1) family with FYVE zinc finger domain
2	Zm00001d002878	Uncharacterized ACR YggU family COG1872 containing protein expressed
2	Zm00001d002879	Unknown
2	Zm00001d002880	Isopropylmalate dehydrogenase 2
2	Zm00001d002881	Putative pentatricopeptide repeat-containing protein
2	Zm00001d002882	SKP1-interacting partner 15
2	Zm00001d002884	Integral membrane protein
2	Zm00001d002885	Putative CCR4-associated factor 1 homolog 3
2	Zm00001d002886	Unknown
2	Zm00001d002887	Unknown
2	Zm00001d002888	MtN21 nodulin protein-like

2	Zm00001d002889	Soluble inorganic pyrophosphatase
2	Zm00001d002890	Unknown
2	Zm00001d002891	Chaperone DnaJ-domain superfamily protein
2	Zm00001d002893	WAT1-related protein
2	Zm00001d002896	TUB-transcription factor 4
2	Zm00001d002897	Peroxidase 14
2	Zm00001d002898	Peroxidase 12
2	Zm00001d002899	Peroxidase 12
2	Zm00001d002900	Unknown
2	Zm00001d002901	Peroxidase 12
2	Zm00001d002902	Peroxidase 12
2	Zm00001d002904	PLC-like phosphodiesterases superfamily protein
2	Zm00001d002905	Protein kinase superfamily protein
2	Zm00001d002906	Unknown
2	Zm00001d002907	RNA polymerase II Rpb4 core protein
2	Zm00001d002912	Glyoxal oxidase-related protein
2	Zm00001d002915	RNA polymerase II Rpb4 core protein
2	Zm00001d002916	Glyoxal oxidase-related protein
2	Zm00001d002917	RNA-binding region RNP-1

2	Zm00001d002919	Tetratricopeptide repeat (TPR)-like superfamily protein
2	Zm00001d002922	Inositol polyphosphate multi kinase beta
2	Zm00001d002925	AP-4 complex subunit mu
2	Zm00001d002928	DUF740 family protein
2	Zm00001d002929	Auxin response factor 17
2	Zm00001d002930	Unknown
2	Zm00001d002933	DDB1- and CUL4-associated factor homolog 1
2	Zm00001d002934	NAC domain-containing protein 30

a) Annotations were obtained from MaizeGDB databases (http:// www.maizegdb.org).

Supplementary Table S2. Maize ear traits of lx9801 and sub-$CSSL_{16}$ in two environments.

Traits	Environments	Parents		
		lx9801	sub-$CSSL_{16}$	P-value
Ear width(mm)	E1	48.38±1.44	48.44±1.04	9.42E-01
	E2	46.84±3.05	47.01±2.01	9.22E-01
Ear length(cm)	E1	11.86±2.34	11.95±1.66	8.06E-01
	E2	12.03±2.79	12.18±1.14	5.79E-01
Kernel row number	E1	12.40±0.89	13.20±1.09	2.42E-01
	E2	13.20±0.89	12.80±1.10	5.80E-01
Kernel number per row	E1	24.00±2.65	22.80±1.30	3.90E-01
	E2	23.40±2.07	23.00±1.08	7.40E-01
Ear weight(g)	E1	87.45±2.46	83.63±5.94	2.79E-01
	E2	90.42±1.91	91.04±4.85	7.97E-01

E1 and E2 are Xinxiang 2015, Changge 2015, China, respectively.

*, ** indicate significant differences at the 0.05 and 0.01 probability level.

.

Supplementary Table S3. The heterotic effects analysis for *hlEW2b* on ear width trait in the testcross of Zheng58 × sub-CSSL16.

Traitsa	Mean ±SD			d/ab	Mode of effectc
	sub-CSSL16	Zheng58	Zheng58 × sub-CSSL16		
Ear width (mm)	48.44 ±1.04	48.58 ±0.9	52.7 ±1.30	59.86	OD

a) Phenotype of ear width was collected in Xinxiang 2015.

b) The ratio of the estimated dominance to the absolute value of additive effect (d/a, degree of dominance) >1, =1, <1 were regarded as exhibiting overdominance (OD), dominance (D) and partial-dominance (PD), respectively.

c) Mode of genetic effect manifestation.

Supplementary Table S4. Molecular markers developed for fine mapping of *hlEW2b* on chromosome 2.

Marker	AGP v4 position	Forward Primer	Reverse Primer
umc1555	25121821	ATAAAACGAACGACTCTCTCACCG	ATATGTCTGACGAGCTTCGACACC
7HY-304	25177782	ACGTGGTCAATTGCACAGAC	TGTCGCGCTTGTAACCAAAA
6HY-23	25651399	GGGAAGGAGTGGAGGTGGT	TGCATACCCGACTAGCAGTTC
2HY-93	26045713	CTACCTCTTCGCCTCCGC	CCATTTCGCTTTTGCCCT
1HY-100	27934745	GTAGTTTGCTGCTTTTTTGTGTT	CTTGATTCGCTCTCTTAGTTCCT
1HY-114	28088080	CCCGCTTAGGTATCTATCTGC	ATCTTCTGGTTCTGCCGTGA
1HY-123	28153007	CAAATCCAGAAGGAAGAAAAACT	TAGAACGGAGAGGAGCACAAG
bnlg1064	28258664	CTGGTCCGAGATGATGGC	TCCATTTCTGCATCTGCAAC
1HY-146	29463038	CACGGGATTGTGGTAGTAAAAG	TTCTGGAGGTTGTTATTTGCC
1HY-152	29467416	GAGAACCAAAACGGCAAGAA	AGCGGACTACTGCTGACCAT
umc1024	30533873	CCTTTTTCGCCTCGCTTTTTAT	TCGTCGTCTCCAATCATACGTG
Chr2.04-A29	32857673	CCTTTGCTGCTTTGCGACT	GCGTTGGGACCCTACTCG
Chr2.04-A74	33353765	GACCCCCAACAAACCAGTG	CACTTCCCCTCCGTCCC
umc1465	39701635	GTACTTCAAAGCACATGAATGCGA	AGTCTCGACTGCCAATTTACCAAA

Supplementary Table S5. Primers used for detecting relative gene expression level.

Gene ID	Forward Primers	Reverse Primers
Zm00001d011699	GCCTGTGGCCATTCAATTAC	GGTACCAAAGCAGGAGACGA
Zm00001d013140	AGTACATGCGAATGCGACAG	CACATCCTTGACGGTGATGA
Zm00001d034023	CACAGGCCCAGAATTTGATT	TGAAATTCCACCATCAGCAA
Zm00001d034320	GTTGGAAGAGGACAGGCAAC	AGCTAAAAAGCTGCCCATCA
Zm00001d026685	CCCCGAGGTTACACATTCAG	ACCACCTGACAATGCACAGA
Zm00001d030895	CTTTGAGGAAGCCGAAGAAG	TGTTCCACCGGTAGCAATAAG
Zm00001d041352	GGGATTGCCTCCTCCATC	GCAACTACGGTGATCCCTTG
Zm00001d007094	GCTTTTGGATGGTGTCACAAA	ATTGCCAGAAAGGTCAATGC
Zm00001d003017	GCTTCAAATTCCTGGTGCAT	GCCGCAGACAAGTTAGCAAT
Zm00001d017271	ATTGAGGAGATTGCTTTGAAGA	CTCCTTGGGCAATAGGATCA
Zm00001d007180	CGCTGTTCCCACACTACGAG	GTAGGAGCTCTTGCCGAGGT
Zm00001d029031	GGTGCTGCCCTACTACGAGA	TAGGAGCTCTTGCCGAGGT
Zm00001d022254	ACTGCTTCCAAGGGATCGTC	AGCGCCTCCTTGATGTTGT
Zm00001d008376	CTGGGCCTCACCACTACCT	ACAAAGCTTGCGATCCTCAG
Zm00001d051927	CTTGCTCGAGTCATTCACCA	TTGGCACAATCTCATCAACC
β-Actin	CAATGGCACTGGAATGGT	ATCTTCAGGCGAAACACG
